# *Salmonella* small RNA fragment Sal-1 facilitates bacterial survival in infected cells via suppressing iNOS induction in a microRNA manner

**DOI:** 10.1038/s41598-017-17205-4

**Published:** 2017-12-05

**Authors:** Chihao Zhao, Zhen Zhou, Tianfu Zhang, Fenyong Liu, Chen-Yu Zhang, Ke Zen, Hongwei Gu

**Affiliations:** 1 State Key Laboratory of Pharmaceutical Biotechnology, Nanjing Advanced Institute for Life Sciences, School of Life Sciences, Nanjing University, Nanjing, Jiangsu, 210046 China; 2 Jiangsu Engineering Research Center for MicroRNA Biology and Biotechnology, Nanjing, Jiangsu 210093 China; 3 School of Public Health, University of California at Berkeley, Berkeley, CA 94720 USA

## Abstract

*Salmonella* can hijack host atypical miRNA processing machinery to cleave its small non-coding RNA into a ~22-nt RNA fragment, Sal-1, which facilitates *Salmonella* survival in the infected host. The mechanism through which Sal-1 promotes *Salmonella* survival, however, remains unknown. In the present study, we reported that Sal-1 targets cellular inducible nitric oxide synthase (iNOS) in a miRNA manner, leading to attenuation of host cell iNOS/NO-mediated anti-microbial capacity. First, depletion of Sal-1 in *Salmonella*-infected epithelial cells significantly increased the iNOS level but not the levels of various inflammatory cytokines. Bioinformatics analysis and mutagenesis strategies were consistent with the identification of mRNA of iNOS as a target of Sal-1 in both human and mice. Second, western blot and immunohistochemical analysis confirmed that Sal-1 suppressed iNOS expression *in vitro* and *in vivo*, thus reducing the production of NO. Finally, Sal-1 facilitating *Salmonella* survival through suppressing iNOS induction was confirmed in mouse model by expressing mutated iNOS that is not targeted by Sal-1 in mice colon. In conclusion, our study provides new insight into the pathogenic mechanism of intracellular bacteria to modulate host innate immune response.

## Introduction

As a leading cause of food-borne illness worldwide^1^, *Salmonella* have developed different mechanisms to survive in host cells^2–4^. Recently, we found that *Salmonella* can use Ago2-mediated mammalian miRNA processing machinery to cleave *Salmonella* small RNA fragments into the ~22-nt functional RNAs. Sal-1, one of such 22-nt miRNA-like RNA fragments, facilitated the intracellular survival of invaded bacteria in host cells. However, the mechanism by which Sal-1 promotes *Salmonella* intracellular survival in the infected mammalian cells remains unknown.

It is generally believed that host cells have multiple strategies to defend against bacterial infection^5–7^. As a conserved anti-bacterial signaling pathway, the induction of iNOS for the production of nitric oxide (NO) plays a critical role in mutation of DNA, inhibition of DNA repair, inhibition of protein synthesis, and inactivation various enzymes^7,8^. When cells were infected with bacteria, iNOS would be rapidly activated through STAT3 signal pathway^9^ and then high level of NO would be produced. The induced NO was important in host resistance to the infection of bacteria^10^ and viruses^11^. Although it has been reported that a certain level of cellular NO may be required for activating bacterial replication^12^, it is widely accepted that high level of cellular NO can impair intracellular survival of bacteria^13–15^. Generation of high level of inflammatory cytokines serves as another strategy of cell anti-bacteria, for example, Ye *et al*. showed that interleukin-17 (IL-17) secreted by CD4^+^ T cells could augment host defense against bacterial pneumonia^16^, Godinez *et al*. also reported that intestinal T cells amplifies innate immune responses by producing cytokines that regulate innate immune functions during *Salmonella* infection^14^. However, it remains unknown whether the reduction of iNOS/NO or inflammatory cytokine production serves as a mechanism underlying the facilitation of Sal-1 on *Salmonella* intracellular survival.

In the present investigation, we show that, following infection, *Salmonella* rapidly releases RNA fragments into the cytosol of infected host cells. These small bacterial RNAs recruit host cell Ago2-based miRNA processing machinery for cleavage of bacterial RNA into miRNA-like RNA segments including the aforementioned Sal-1^17^. We explored the function of Sal-1 and identified mRNA of iNOS as the target of Sal-1. Our experiments confirmed that, by suppressing NO production, *Salmonella* are able to survive in host epithelial cells.

## Results

### Sal-1 suppresses host cell iNOS but not other inflammatory cytokines

**Figure 1:**
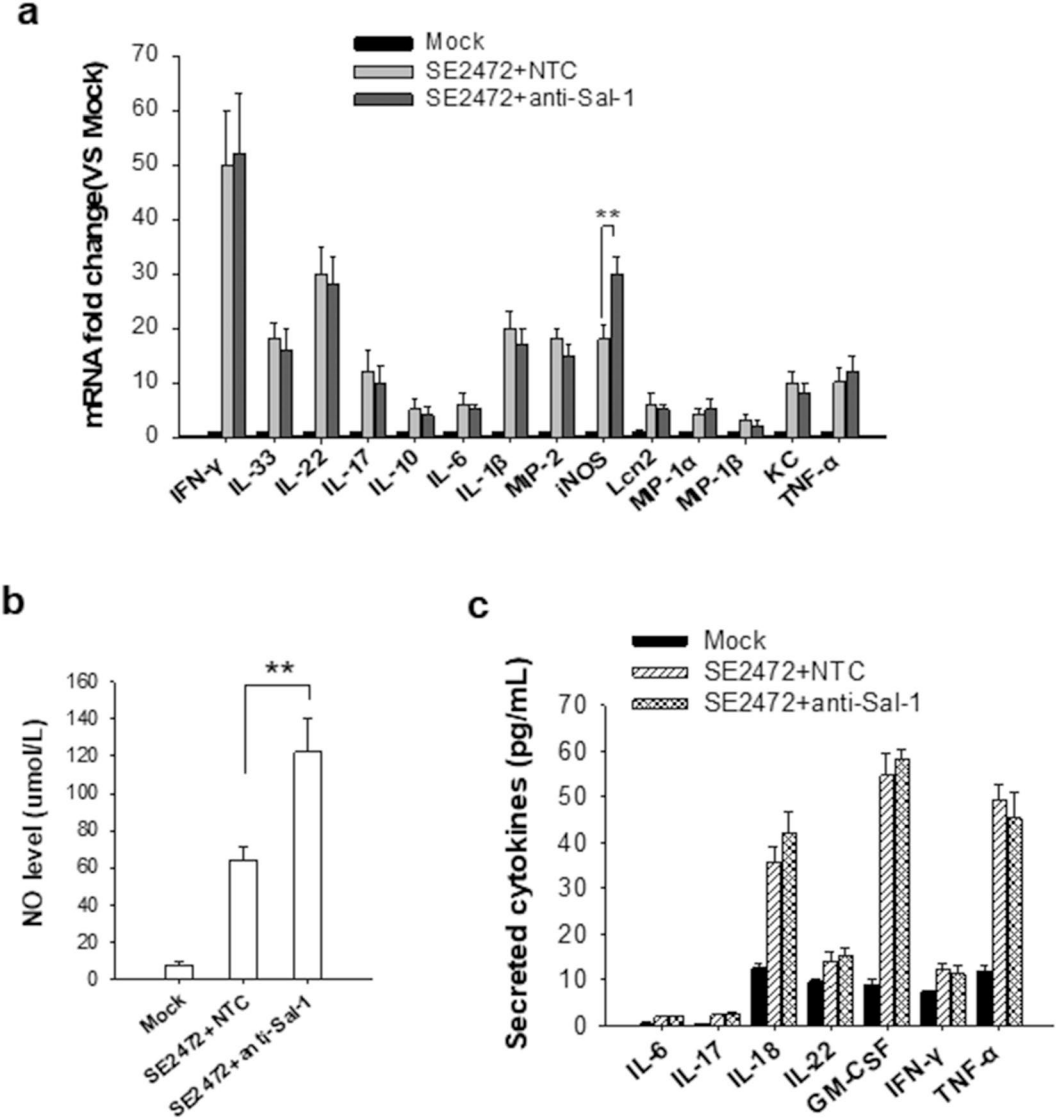
Anti-Sal-1 antagomir treatment promotes iNOS expression and NO production in *Salmonella*-infected host cells. (**a**) mRNA levels of inflammatory cytokines and iNOS in *Salmonella*-infected cells treated with anti-Sal-1 antagomir or NTC. (**b**) NO production in *Salmonella*-infected cells treated with anti-Sal-1 antagomir or NTC. (**c**) Protein levels of cytokines secreted from *Salmonella*-infected cells treated with anti-Sal-1 antagomir or NTC. The data are presented as the mean ± SEM (n = 3). **P < 0.01.

Colon epithelial cells and macrophages are important for regulating the colon immune system’s response to invading *Salmonella*. Host cells produce various inflammatory cytokines such as IFN-γ, IL-1β and IL-6, etc., as well as nitric oxide (NO) as part of anti-bacteria strategy^14,18^. To identify the potential mechanism by which Sal-1 facilitates *Salmonella* infection in human colonic epithelial cells, we compared the inflammatory response of host cells to *Salmonella* infection in the presence or absence of anti-Sal-1 antagomir. Compared to Mock infection, strain SE2472 infection strongly increased the mRNA levels of inflammatory cytokines including IFN-γ, IL-1β, MIP-2, IL-33, IL-17, IL-22, TNFα, etc. (Fig. 1a). However, when *Salmonella*-infected cells were also treated with anti-Sal-1 antagomir, the mRNA levels of most inflammatory cytokines were not altered, whereas the level of iNOS mRNA was further increased compared to *Salmonella*-infected cells treated with control oligonucleotide (NTC) (Fig. 1a). In line with this, measurement of NO production (Fig. 1b) and protein levels of cytokines secreted from *Salmonella*-infected cells (Fig. 1c) also showed that anti-Sal-1 antagomir treatment mainly affected the levels of NO and iNOS in the infected host cells. These results suggest that Sal-1 influences the host immune response by lowering levels of iNOS and NO. As iNOS and its associated NO production serves as a critical anti-microbial mechanism^13,14,19,20^, attenuating NO production in the infected cells may serve as a mechanism by which Sal-1 facilitates bacterial intracellular survival.

### Sal-1 targets human iNOS mRNA

**Figure 2:**
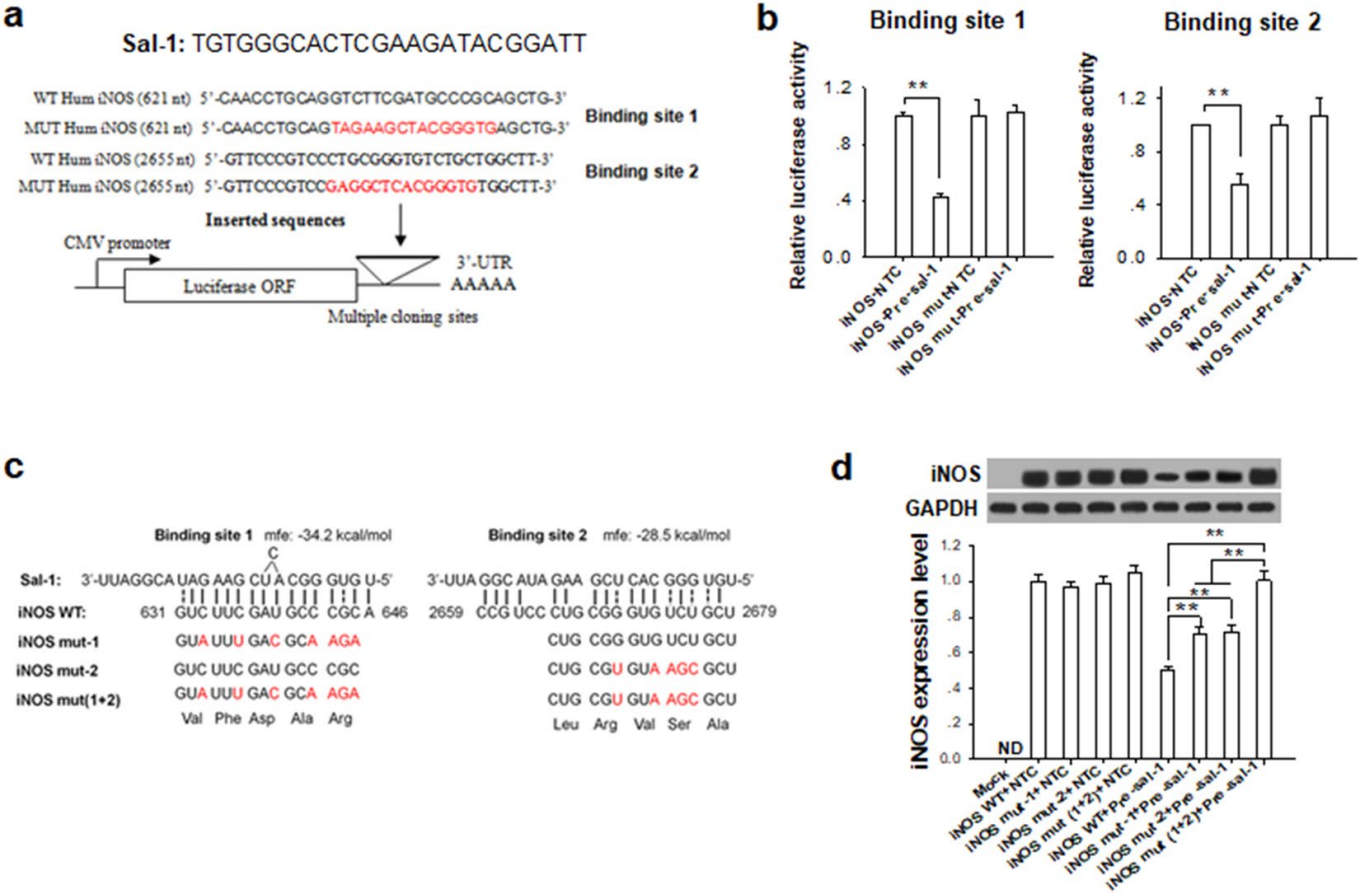
Sal-1 targets human iNOS ORF and suppresses the induction of iNOS by *Salmonella* infection. (**a**) Diagram of the luciferase reporter vector carrying the firefly luciferase-coding sequence attached to wild-type (WT) or mutated (MUT) human iNOS binding sites 1 and 2. (**b**) Relative luciferase activity in HEK-293T cells co-transfected with luciferase reporter vectors containing WT or MUT form of Sal-1-binding sites (1 or 2) and Pre-Sal-1. (**c**) Two putative binding sites for Sal-1 on human iNOS ORF were predicted using RNAhybrid (http://bibiserv.techfak. uni-bielefeld.de/ rnahybrid). Synonymous mutation at two Sal-1 binding sites on iNOS was performed to generate iNOS Mut-1, Mut-2 or Mut (1 + 2) expression vectors. (**d**) Level of iNOS in RAW macrophages co-transfected with WT or Mut iNOS, Pre-Sal-1 or NTC. The data are presented as the mean ± SEM (n = 3). **P < 0.01, ND, not detected.

We next explored the mechanism by which Sal-1 reduces the level of iNOS in host cells. According to our recent study, Sal-1 had a ‘stem’ structure-containing precursor and was processed like a miRNA^17^, we speculated that Sal-1 might function analogously to endogenous miRNAs and suppress iNOS at the post-transcriptional level. Bioinformatics analysis using the miRWalk algorithm^21^ was thus performed to screen potential human genes that could be targeted by Sal-1. We identified the mRNA transcribed from the gene for human iNOS as a highly conserved Sal-1 target gene, with two putative binding sites for Sal-1 in the iNOS mRNA ORF (Fig. 2a, upper panel). The minimum free energy of binding was −34.2 kcal/mol for the binding site 1 (BS1) and −28.5 kcal/mol for the binding site 2 (BS2), respectively. The direct binding of Sal-1 to the BS1 and BS2 in iNOS mRNA ORF was further validated by luciferase reporter assay. Wild-type (WT) or mutated (MUT) Sal-1 binding regions (containing BS1 or BS2) of human iNOS were inserted into a luciferase reporter plasmid (Fig. 2a, lower panel) and then co-transfected with Pre-Sal-1 into HEK-293T cells. Pre-Sal-1 transfection significantly reduced luciferase activity of WT reporter but not MUT reporter (Fig. 2b). To determine whether Sal-1 could directly target iNOS mRNA, we used site-directed mutagenesis to generate various iNOS constructs in which the Sal-1 target sites were mutated but the iNOS amino acid sequence remained unchanged, including iNOS Mut-1 (BS1 mutated), Mut-2 (BS2 mutated) and Mut (1 + 2) (both BS1 and BS2 mutated) (Fig. 2c). These constructs were co-transfected with Pre-Sal-1 or NTC (control) into RAW264.7 macrophages. As expected, Sal-1 strongly downregulated the expression of WT iNOS (Fig. 2d). However, when both Sal-1 target sites on iNOS ORF were mutated, Sal-1 had no effect on the expression of iNOS.

### Sal-1 inhibits iNOS expression and NO production in a miRNA manner

**Figure 3:**
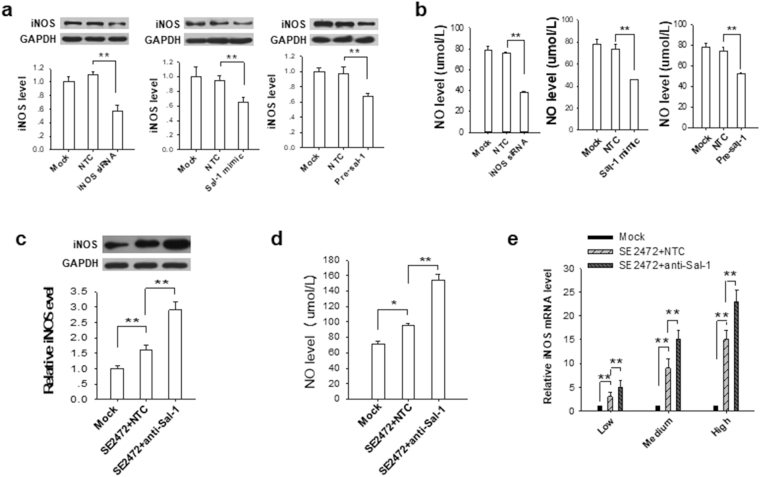
Inhibition of iNOS induction and NO production in human colonic HT-29 cells by Sal-1. (**a**) Downregulation of iNOS by transfection with iNOS siRNA, Sal-1 mimic and Pre-Sal-1. (**b**) Inhibition of NO production by transfection with iNOS siRNA, Sal-1 mimic and Pre-Sal-1. (**c**) Upregulation of iNOS in SE2472-infected HT-29 cells by transfection with anti-Sal-1 antagomir. (**d**) Enhancement of NO production in SE2472-infected HT-29 cells by transfection with anti-Sal-1 antagomir. (**e**) Sal-1 antagomir increased iNOS mRNA level in HT-29 cells directly infected with various dose of strain SE2472. The data are presented as the mean ± SEM (n = 3). *P < 0.05. **P < 0.01.

The regulatory role of Sal-1 in host cell iNOS expression was further validated in human colonic HT-29 cells. We transfected LPS-treated-HT-29 cells with Sal-1 mimic or Pre-Sal-1 or iNOS siRNA and then assessed the inhibition of Sal-1 on iNOS expression. Consistent with our hypothesis that Sal-1 targets iNOS mRNA, the expression of iNOS protein in LPS-treated HT-29 cells was markedly reduced when transfected with the Sal-1 mimic or Pre-Sal-1 (Fig. 3a). The level of NO in LPS-treated HT-29 cells was also markedly reduced by Sal-1 mimic or Pre-Sal-1 transfection (Fig. 3b). In term of NO production, overexpression of Sal-1 mimics the phenotype of transfection with iNOS siRNA. Moreover, we pre-treated HT-29 cells with or without Sal-1 antagomir and then infected the cells with *Salmonella*. As shown in Fig. 3c and d, *Salmonella* infection increased iNOS expression (Fig. 3c) and NO level (Fig. 3d) in HT-29 cells compared to Mock infection. However, when Sal-1 was depleted by Sal-1 antagomir, the levels of iNOS expression (Fig. 3c) and NO (Fig. 3d) in *Salmonella-*infected intestinal epithelial cells were further increased. These results support the inhibitory effect of Sal-1 on the expression of iNOS in host cells. We also detected the levels of iNOS mRNA in HT-29 cells after infection with *Salmonella* SE2472 (low, MOI = 1; medium, MOI = 10; and high, MOI = 100). As shown in Fig. 3e, Sal-1 antagomir significantly increased iNOS mRNA level in HT-29 cells infected with various dose of *Salmonella* SE2472, suggesting that Sal-1 might be responsible for degradation of iNOS mRNA, analogous to the function of host miRNA on its target genes.

**Figure 4:**
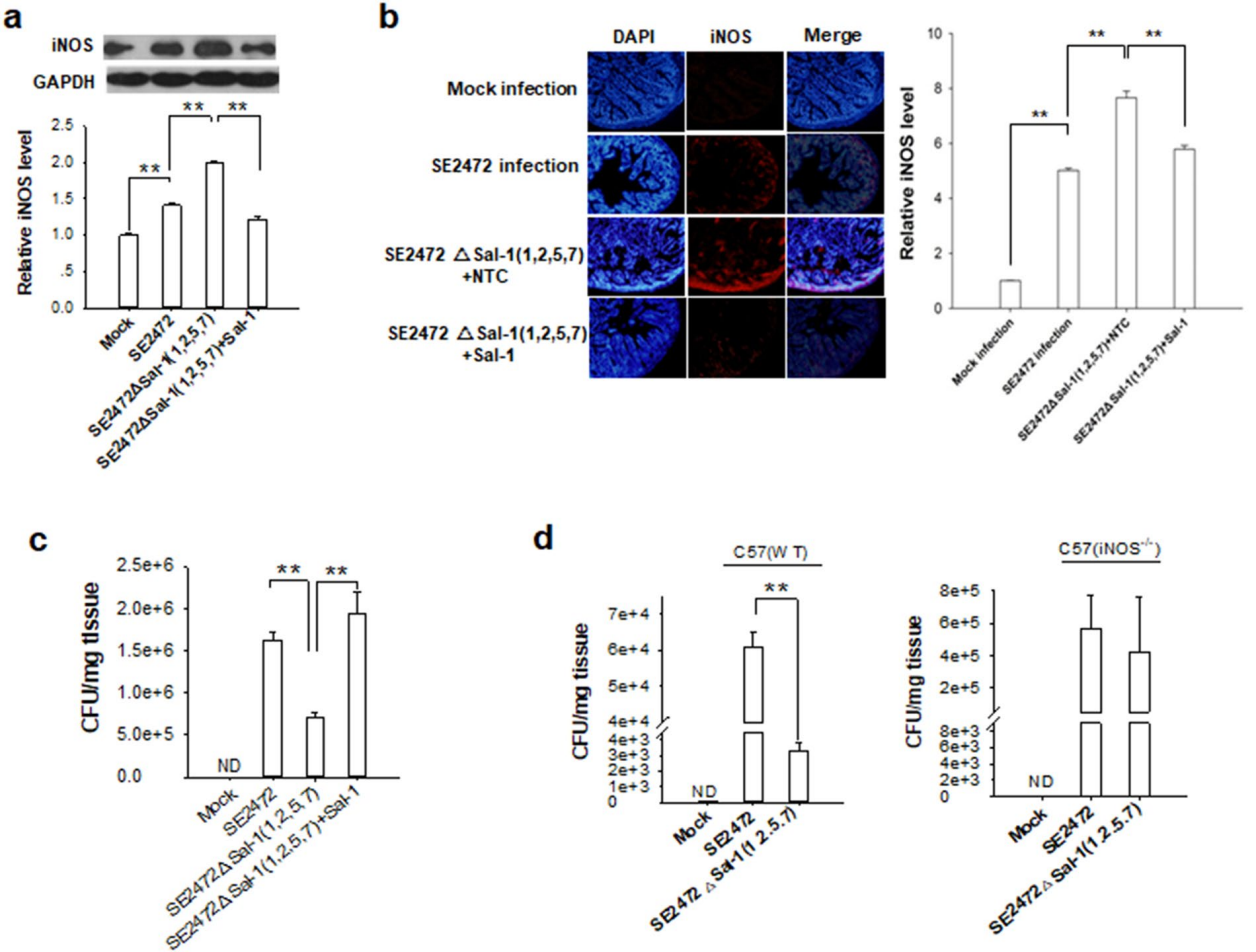
Deletion of Sal-1 copies in bacterial genome increases iNOS expression and impairs the intracellular survival of SE2472 strain. (**a**,**b**) Infection with SE2472∆Sal-1(1,2,5,7) strain resulted in higher level of iNOS in HT-29 cells (**a**) and mouse colon tissues (**b**) than that with SE2472 or Mock infection. (**c**,**d**) The survival rate of SE2472 strain and SE2472∆Sal-1(1,2,5,7) strain in WT mice (c and d, left panel) and iNOS^−/−^ mice (d, right panel). Note that the defect of SE2472∆Sal-1(1,2,5,7) on attenuating iNOS expression and enhancing intracellular survival can be largely recovered by transfection with Sal-1 mimic. The data are presented as the mean ± SEM (n = 3). **P < 0.01. ND, not detected.

Furthermore, a mutated *Salmonella* SE2472 strain, in which Sal-1 sites were deleted, was generated to confirm the regulation of the iNOS mRNA by Sal-1. We successfully deleted four Sal-1 sites in SE2472 strain to generate a mutated strain SE2472ΔSal-1(1,2,5,7), without affecting the normal bacterial growth rate^17^. Infection with SE2472 mutant in HT-29 cells resulted in a higher iNOS level compared to the infection with wild-type (WT) SE2472 (Fig. 4a). However, the defect of SE2472 mutant in controlling the excessive iNOS expression and promoting bacterial intracellular survival could be largely restored by increasing cellular Sal-1 level through forced expression of Sal-1 mimic. Next, we infected mice with WT SE2472 or SE2472 mutant and then examined the effect of Sal-1 on iNOS induction and bacterial survival. As shown in Fig. 4b, iNOS level in mouse intestine infected with SE2472∆Sal-1(1,2,5,7) was higher than that in SE2472-infected mice. The increase of iNOS level in mice infected with SE2472∆Sal-1(1,2,5,7) was abolished by co-administration with Sal-1. Bacterial survival assay confirmed that the survival rate of SE2472∆Sal-1(1,2,5,7) in mouse intestine was significantly lower than WT SE2472, while increase of Sal-1 level via co-administration of Sal-1 markedly enhanced the survival rate of SE2472 mutant (Fig. 4c). Finally, we established an infection model in iNOS-deficient (iNOS^−/−^) mice and tested bacterial survival rate. WT mice and iNOS^−/−^ mice were infected with WT SE2472 or SE2472 mutant, and the respective bacterial survival rate in these mice was assessed. The survival rates of both WT SE2472 and SE2472 mutant in iNOS^−/−^ mouse group were higher than those in WT mice group, suggesting that iNOS played an important role in controlling *Salmonella* infection (Fig. 4d). However, the survival rate of SE2472 mutant was markedly lower than WT SE2472 in the intestine tissues of WT mice (Fig. 4d, left panel), while there is no significant difference between WT SE2472 and SE2472 mutant in iNOS^−/−^ mice group (Fig. 4d, right panel). Taken together, these results showed that Sal-1 suppresses the expression of iNOS, thus facilitating the survival of *Salmonella* during infection.

### Sal-1 enhances *Salmonella* infection in mice via suppressing colonic epithelial iNOS

**Figure 5:**
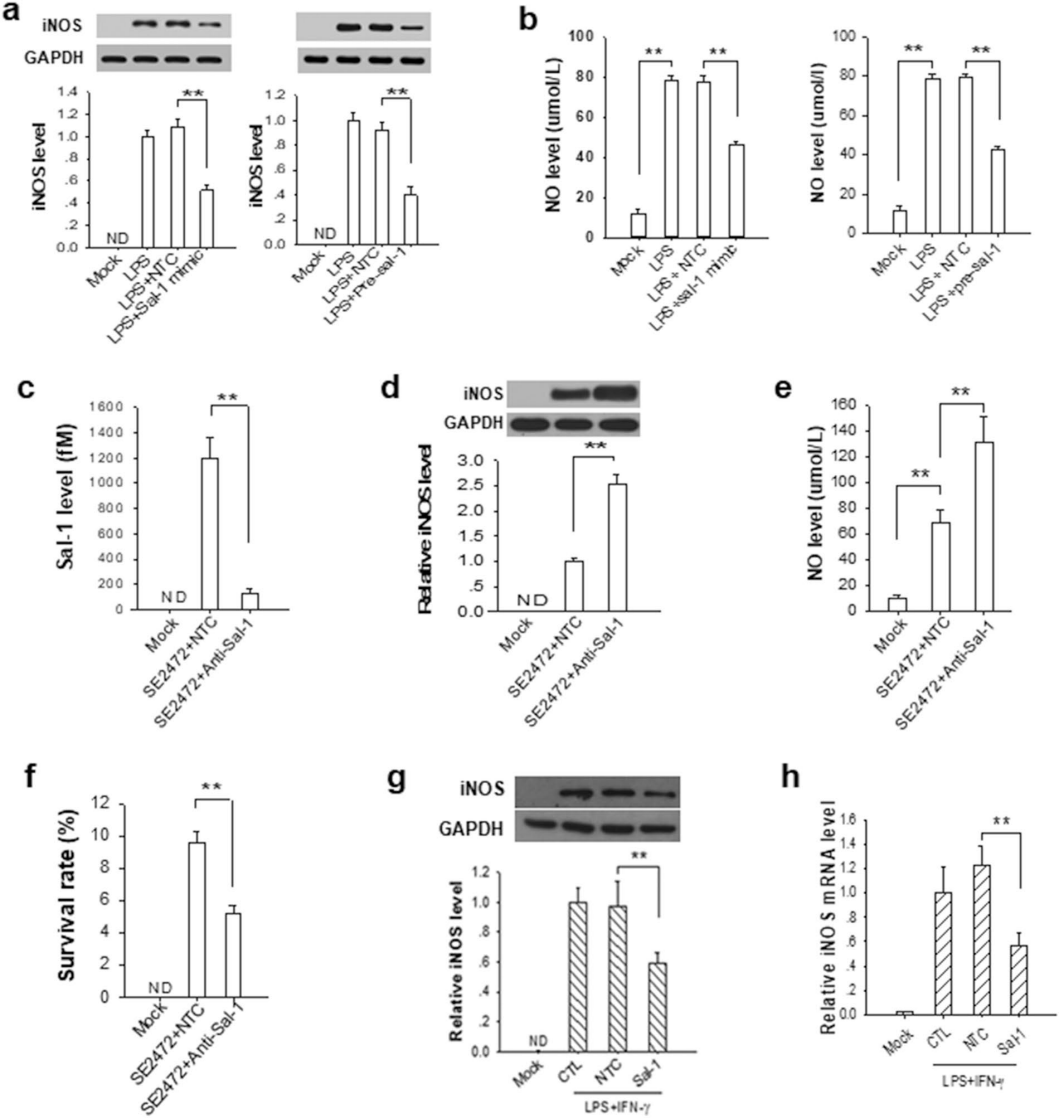
Role of Sal-1 in decreasing the levels of iNOS mRNA, protein and NO in RAW264.7 macrophages (**a**–**f**) and mouse primary bone marrow derived macrophages (BMDMs) (**g**,**h**) during *Salmonella* infection. (**a**) Relative iNOS level in RAW264.7 cells with or without LPS stimulation. LPS-stimulated RAW264.7 cells were also transfected with Sal-1 mimic, control oligonucleotides (NTC), Pre-Sal-1 or Pre-ncRNA, respectively. (**b**) Relative level of NO in LPS-stimulated RAW264.7 cells. (**c**–**f**) Levels of Sal-1 (**c**), iNOS (**d**) and NO (**e**), and bacterial intracellular survival rate (**f**) in *Salmonella*-infected RAW264.7 cells. The infected RAW264.7 cells were also transfected with Sal-1 antagomir or NTC. (**g**,**h**) Relative iNOS protein (**g**) and mRNA (**h**) level in mouse primary BMDMs with or without LPS and IFN-γ stimulation. LPS and IFN-γ-stimulated BMDMs were also transfected with Sal-1 mimic or control oligonucleotides (NTC), respectively. The data are presented as the mean ± SEM (n = 3). **P < 0.01. ND, not detected.

We next evaluated the process through which Sal-1 suppresses iNOS expression in a mouse model of *Salmonella* infection. First, because the homology is only 82.4% between human iNOS and mouse iNOS, we used the RNAhybrid software to examine the binding sites of Sal-1 on mouse iNOS mRNA. As shown in Supplementary Fig. 1a, the mouse iNOS ORF also has two potential binding sites for Sal-1. After mutating each binding site and testing the binding of Sal-1 to mouse iNOS ORF using luciferase reporter assay, we confirmed that Sal-1 strongly bound to the binding site 1 and 2 (Supplementary Fig. 1b). Then we demonstrated the impact of Sal-1 on mouse iNOS expression in RAW 264.7 cells and primary mouse bone marrow derived macrophages (BMDMs). As expected, transfection of RAW264.7 macrophages with Sal-1 mimic or Pre-Sal-1 markedly reduced mouse iNOS expression (Fig. 5a) and NO level (Fig. 5b) compared to cells transfected control oligonucleotides. In a similar fashion, we infected RAW 264.7 cells with *Salmonella* SE2472 and then co-transfected them with the Sal-1 antagomir. As shown, Sal-1 antagomir significantly depleted Sal-1 in SE2472-infected cells compared to cells infected with SE2472 without Sal-1 antagomir (Fig. 5c). Sal-1 antagomir also markedly increased the levels of both iNOS expression (Fig. 5d) and cellular NO production (Fig. 5e). As the consequence, *Salmonella* intracellular survival rate in RAW 264.7 macrophages was reduced in the cells in which the antagomir had been transfected (Fig. 5f). We next performed experiments employing murine BMDM. BMDMs were treated with LPS and IFN-γ to induce iNOS expression, then transfected with or without Sal-1 mimic. As shown in Fig. 5g and h, the expression of iNOS was successfully induced with LPS and IFN-γ, as evidenced by higher levels of iNOS in stimulated versus unstimulated cells. Both protein and mRNA levels of iNOS in Sal-1-treated group were markedly reduced compared to the cells without Sal-1 transfection.

**Figure 6:**
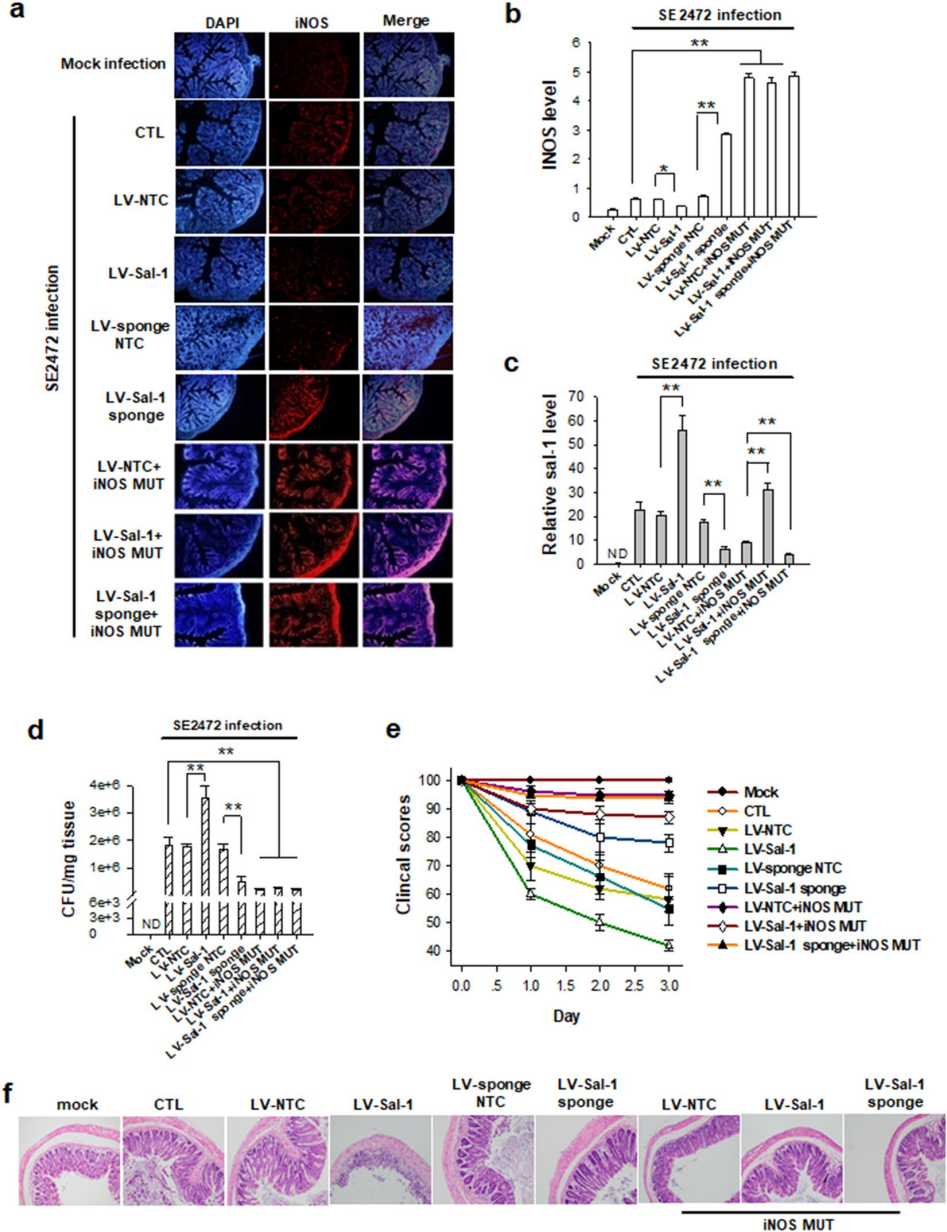
Role of Sal-1-targeting iNOS in facilitating *Salmonella* infection in mice. Mouse iNOS gene was mutated at two Sal-1 binding sites through synonymous mutation and was inserted into lentivirus vectors for overexpressing mutated iNOS. Pre-Sal-1 was inserted into lentivirus vectors to overexpress Sal-1. A lentivirus-based molecular sponge for Sal-1 was also generated. Female BALB/c mice (6–8 weeks, 10 mice per group) were inoculated intragastrically with *Salmonella*, and the lentiviruses were slowly administered into the lumen of mouse colon via a catheter prior to *Salmonella* infection. (**a**) The iNOS level in mouse colon tissues analysed through immunofluorescence labelling. (**b**) The mean fluorescence intensity of iNOS in mouse colon tissue was determined using software Image Pro Plus 6.0. (**c**–**e**) Sal-1 levels (**c**), *Salmonella* bacteria count (**d**) in mouse colon tissues and the clinical scores of the mice (**e**) after infected with *Salmonella*. (**f**) Representative images of H&E staining showed the damages of colon tissue in mice treated with *Salmonella*. The data are presented as the mean ± SEM (n = 3). *P < 0.05. **P < 0.01. ND, not detected.

Given that mouse iNOS ORF has two Sal-1 binding sites, we created an iNOS expression construct in which these two target sites of Sal-1 were mutated via site-directed mutagenesis (Supplementary Table 1). This mutated construct (iNOS MUT) was then inserted into lentivirus vectors (LV-iNOS MUT) to allow the expression of functional iNOS which was not vulnerable to inhibition by Sal-1. Besides the Sal-1 expressing lentivirus (LV-Sal-1), we also constructed a lentivirus vector containing multiple sequence repeats that are completely complementary to Sal-1 thereby creating a molecular sponge to sequester Sal-1 (LV-Sal-1 sponge). To test the effect of Sal-1-targeting iNOS on *Salmonella* infection in mice, we directly delivered either LV-iNOS MUT, LV-Sal-1 or LV-Sal-1 sponge (each 5 × 10^7^ TU) into the lumens of female BALB/c mouse colon using the method described previously^22^. Mice were then intragastrically injected with strain SE2472 (6 × 10^5^ CFU/ml) on the fourth day. On day 3 post-infection, mice were sacrificed and the iNOS levels in serum and colon tissues were assayed by immunofluorescence analysis and ELISA. As shown in Fig. 6a and b, the level of iNOS was markedly increased in mouse colon tissues by strain SE2472 infection relative to uninfected mice (Mock group). Targeting iNOS mRNA by lentivirus-delivered Sal-1 (LV-Sal-1) significantly reduced the level of iNOS relative to mice infected with SE2472 alone. Finally, in the mice receiving the LV-Sal-1 sponge, the levels of iNOS were increased relative to mice that were infected with SE2472 alone. As expected, when the mice were administered with LV-iNOS MUT, which is not vulnerable to suppression by Sal-1, iNOS level in the mouse colon tissues remained high and was not influenced by LV-Sal-1 or LV-Sal-1 sponge. These results suggested that Sal-1 could target iNOS and suppress its expression in mouse colon tissues during *Salmonella* infection.

To explore whether Sal-1 inhibited iNOS expression after infection could facilitate *Salmonella* survival during the infection, we next assayed Sal-1 levels in mouse colon tissues (Fig. 6c). Compared with non-infected mice, *Salmonella*-infected mice displayed a higher Sal-1 level in colon tissues, with the highest Sal-1 level in the group co-administered with lentivirus-delivered LV-Sal-1. In contrast, Sal-1 level in SE2472-infected mouse colon tissues was markedly decreased by administering with LV-Sal-1 sponge. Interestingly, when *Salmonella*-infected mice were administered with LV-iNOS MUT, the level of Sal-1 was significantly decreased compared to the mice infected with *Salmonella* alone. Therefore, we evaluated *Salmonella* colonization in mouse colon. As shown in Fig. 6d, *Salmonella* colonies were detected in all *Salmonella*-infected mice. The CFU counts in the mice infected with SE2472 were significantly increased by LV-Sal-1 but decreased by LV-Sal-1 sponge. Administration of LV-iNOS MUT significantly reduced the *Salmonella* counts in the mice infected with strain SE2472. We next divided mice into two groups with one group receiving LV-iNOS MUT and LV-Sal-1 and the other group receiving LV-iNOS-MUT and LV-Sal-1 sponge. The data showed that the inhibition of LV-iNOS MUT on *Salmonella* colonization was not affected by LV-Sal-1 or LV-Sal-1 sponge treatment. The results suggested that LV-iNOS MUT expression can suppress bacterial intracellular survival through increasing the cellular level of NO.

Next, we assessed the disease degree in BALB/c mice caused by *Salmonella* infection using a clinical scoring system previously described^17^. As shown in Fig. 6e, Mock infection mouse group got the highest 100 scores and the mice were completely healthy. The mice administered *Salmonella* SE2472 with or without control lentivirus vector (SE2472, SE2472 + LV-NTC) both displayed certain illness and their score was around 60. However, the mice infected with strain SE2472 and also administered with LV-Sal-1 (SE2472 + LV-Sal-1) developed a much severe illness with score below 45. In contrast, the illness burden caused by *Salmonella* infection, however, was able to be mitigated by using LV-Sal-1 sponge to deplete cellular Sal-1 (SE2472 + LV-Sal-1 sponge). When the *Salmonella*-infected mice were co-administered with LV-iNOS MUT that expresses functional iNOS which cannot be repressed by Sal-1, mice almost completely cleared the bacterial infection and the symptoms of *Salmonella* infection almost disappeared. In addition, the colon microstructure changes detected by H&E staining (Fig. 6f) were consistent with the clinical score evaluation. Taken together, these results suggested that Sal-1 enhances *Salmonella* infection in mouse colonic epithelial cells through suppressing the epithelial cell iNOS expression.

## Discussion

In the current study, we show that *Salmonella*-encoded ~22-nt Sal-1 facilitates intracellular survival of *Salmonella* through targeting host cell iNOS mRNA, leading to downregulation of iNOS and decrease of anti-bacterial NO production.

RNA molecules encoded by viral or non-viral pathogens have been shown to act as regulators in eukaryotic cells^23,24^. Early work by Fraser *et al*. showed that *E*. *coli* double-stranded RNA could silence genes in the *C*. *elegans* repertoire^24^. Non-pathogenic *E*. *coli* were thus used to deliver shRNA fragments into animal cells^25^. Application of deep RNA sequencing and tiling array have shown that bacteria contain large amount of non-coding RNAs^26,27^. Besides executing their regulatory function in the bacteria, bacterial noncoding RNAs can also regulate *C*. *elegans* gene expression^28^. Recently Weiberg *et al*.^29^ showed that “virulent” sRNA effectors derived from fungal pathogens could be transferred into host to suppress immunity thereby establishing infection. Consistent with bacterial products interfering with host expression of proteins, our results show that after processing by host cell Ago2 complex, the small RNA products produced by *Salmonella* bacteria can interfere with the expression of host iNOS. Specifically, *Salmonella*-encoded Sal-1 acts in an miRNA manner, binding to iNOS mRNA and diminishing the iNOS expression and the NO production. Identification of such functional bacterial small RNA fragments in the infected host cells expands the role of bacterial small RNAs in suppressing the host cell immune response.

Previous studies showed that *Salmonella* infection induced robust iNOS expression and iNOS-dependent NO and its congeners. Moreover, NO contributed to host control of *Salmonella* infections^15^. Our results are consistent with NO being a major weapon inhibiting colonization by *Salmonella*. We observed that host expression of iNOS and production of NO rapidly increased in host cells and in the mouse colon after infection by *Salmonella* even in the presence of Sal-1. When the Sal-1 was knocked out, cellular iNOS expression and NO level were further increased and *Salmonella* proliferation became far less robust. Our findings are thus consistent with the view that high levels of NO are deleterious for *Salmonella* bacteria^13–15^. Intriguingly, other groups have reported that NO production does relatively little harm to *Salmonella* ^30–32^, because the *Salmonella* enzyme, nitric oxide denitrosylase, can rapidly metabolize NO^33^. There is even suggestion in the literature that the relationship between *Salmonella* growth and ambient NO levels in curvilinear. A minimal level of NO is necessary for promoting robust growth of *Salmonella* by enabling nitrate respiration^12^. However, as previously stated, high levels of NO are deleterious to *Salmonella* bacteria. Thus, *Salmonella* exploits many strategies to maintain optimal levels of NO during various phases of the infection^32^. Rather than totally eliminating ambient NO, Sal-1 function might be properly characterized as establishing the optimal level of NO for promoting bacterial growth. Our results provide a novel insight to increase our understanding of *Salmonella* pathogenesis.

In conclusion, the present investigation shows that *Salmonella*-encoded miRNA-like fragment Sal-1 facilitates *Salmonella* survival in the infected host cells through downregulating the cellular iNOS and suppressing NO overproduction. Our study provides the first evidence that small functional RNA fragments like Sal-1 may play a significant role in promoting bacterial infection and intracellular survival.

## Materials and Methods

### Cells, reagents, antibodies and bacteria strains

Human intestinal epithelial cells HT-29 were obtained from the China Cell Culture Centre (Shanghai, China). Human HEK-293T cells and mouse RAW264.7 macrophages were purchased from American type culture collection (ATCC). HT-29 cells were cultured in RPMI-1640 medium (Gibco, Carlsbad, CA) supplemented with 10% FBS (Gibco) and 1% Penicillin-Streptomycin (Gibco). HEK-293T and RAW264.7 cells were cultured in DMEM supplemented with 10% FBS. All cells were maintained in a humidified incubator at 37 °C with 5% CO_2_. The iNOS antibody (#2977) was purchased from Cell Signal Technology (Danvers, MA). The Nitric Oxide Synthase Detection System (Fluorimetric) was purchased from Sigma-Aldrich (St Louis, MO). The mouse monoclonal anti-GAPDH antibody was purchased from Santa Cruz Biotechnology (Santa Cruz, CA). Highly virulent *Salmonella* SE2472 strain (LD50 < 10^3^ organisms)^34^, as well as *E*. *coli* strains Top 10 and DH5α were grown in Luria-Bertani broth.

### Plasmid construction and luciferase reporter assay

A mammalian expression vector encoding the human or mouse iNOS ORF (pReceiver-M02-iNOS) was obtained from GeneCopoeia (Germantown, MD). Primers were designed to mutate the Sal-1 target site in the human iNOS coding region without altering the amino acid sequence of iNOS. For Mut1, site 1 (VFDARS) was mutated as follows: before mutagenesis, GTC TTC GAT GCC CGC; after mutagenesis, GTa TTt GAc GCa aGa. For Mut2, site 2 (RVS) was mutated as follows: before mutagenesis, CGG GTG TCT; after mutagenesis, CGt GTa agc. For Mut (1 + 2), both sites 1 and 2 were simultaneously mutated as described above. The mouse iNOS mutation site 211–228 (VTSTRP) was mutated as follows: before mutagenesis, GTG ACA TCG ACC CGT CCA; after mutagenesis, GTt ACA TCg ACC CGa CCt. Site 313–336 (KSKSCLGS) was mutated as follows: before mutagenesis, AAG TCC AAG TCT TGC TTG GGG TCC; after mutagenesis, AAa TCg AAG TCa TGC ctt GGG agt. Site 2653–2658 (VP) was mutated as follows: before mutagenesis, GTG CCC; after mutagenesis, GTc CCg. Site 3016–3033 (LVFGCR) was mutated as follows: before mutagenesis, TTG GTG TTT GGG TGC CGG; after mutagenesis, cTG GTt TTc GGc TGt aGG. The mutated mouse iNOS gene was inserted into a lentivirus vector to produce a recombinant lentivirus (Lenti-iNOS-mut). To generate luciferase reporters, the amplified fragments containing iNOS sites 1 and 2 were each cloned into the 3′-UTR region of the pMIR-reporter plasmid (Ambion, Austin, TX). Sequencing was performed to confirm the efficient insertion. For luciferase reporter assays, equal amounts (20pmol) of Pre-Sal-1, Sal-1 mimic, or scrambled negative control RNA was co-transfected with 0.2 μg of firefly luciferase reporter plasmid into cells in 24-well plates. The β-galactosidase vector (Ambion, 0.1 μg) was also transfected into cells as a transfection control. Cells were collected and analysed using a luciferase assay kit (Promega, Madison, WI) at 24 h post-transfection. To generate the Sal-1 sponge element, we introduced four copies of complementary sequence to Sal-1 into the 3′-UTR of enhanced green fluorescent protein (EGFP) and inserted them into a pGLVH1/GFP vector. The sequences were then packaged into a lentivirus. For Sal-1 overexpression, the pre-Sal-1 hairpin structure was inserted into a pGLVH1/GFP vector and then packaged into a lentivirus.

### Nitric oxide detection

The nitric oxide detection kit (Enzo, Farmingdale, NY) was used for the quantitative determination of NO in cell culture supernatants. The detection process was in accordance with the manufacturer’s instructions. Briefly, samples collected from different treatments were diluted at 1:2 in Reaction Buffer and filtered through a 10,000 MWCO filter (Millipore, Burlington, MA). The processed supernatants were used directly in the assay according to the manufacturer’s instruction and the optical density (OD) at 540–570 nm was detected using the microplate reader. The total NO concentration was calculated using the standard curve established using Nitrate Standard solution.

### H&E Staining

The colon tissues were collected from different treated mice and subjected to H&E staining to evaluate the tissue damages. H&E staining was performed according to routine protocols. The slides were then examined and photographed using Olympus BX51 microscope (Tokyo, Japan).

### Immunohistochemical analysis

For the *in vivo* infection studies, the mice were euthanized at 3 days after the *Salmonella* infection. Briefly, the colon tissues were harvested, embedded in optimal cutting temperature compound, frozen, cut into 5–7 μm thick sections, and fixed with 10% buffered formalin. The presence of iNOS in the colon tissues was investigated using a polyclonal rabbit iNOS antibody (BD Transduction Laboratories). The nuclei were stained with 4,6-diamidino-2-phenylindole (DAPI; Sigma). The samples were examined under a fluorescence microscope.

### Bioinformatics analysis

The Sal-1-target sites in CDS or UTR of the transcripts were identified by using two criteria to determine the match between the transcript and Sal-1. The first criterion for target recognition, referred to as the “seed rule”, was base pairing between the “seed region” (a conserved core sequence which was mostly situated at positions two to eight from the 5′-end of the mature miRNA) and the target^35^. Second, the free energy of the hybrid was within the range of authentic miRNA-target pairs (usually less than -25 kcal/mol).

### *In vivo* studies

All animal experimental protocols were in accordance with the National Institutes of Health Guidelines for the Use of Experimental Animals and approved by Nanjing University Animal Care Committee (Nanjing, China). 6–8 weeks old female BALB/c mice were randomly divided into 10 mice per group, each group was treated differently according to the experimental design, and the Mock infection group was inoculated with PBS. To assay the regulatory relationship between Sal-1 and iNOS in *Salmonella* infection, lentiviruses overexpressing Sal-1 or absorbing Sal-1 were used. Briefly, 0.5–2 × 10^8^ TU lentivirus was administered into the lumen of mouse colons via a catheter^17^. To further validate iNOS expression regulated by Sal-1, we also administered mice with lentivirus LV-iNOS WT or LV-iNOS MUT that expresses mouse iNOS but has mutated Sal-1 binding sites. Three days after lentivirus administration, each mouse was infected with 5 × 10^6^ CFU of *Salmonella* SE2472 intragastrically and the clinical features were monitored during bacterial infection. To evaluate the mouse disease following *Salmonella* infection, a clinical scoring system previously described was used with minor modifications^36^. The mice were sacrificed on day 3 post-inoculation with *Salmonella*, and Sal-1 expression, *Salmonella* colonies and iNOS expression in mouse colons were determined. The *Salmonella* survival rate was also assessed by counting the number of *Salmonella* colonies in mouse colon tissue homogenates using *Salmonella* detection CHROMagar^TM^ plates^17^.

### Statistical analysis

The luciferase reporter assays were repeated five times, and each experiment was performed in triplicate. The data are presented as the mean ± SEM of at least three independent experiments. The difference with *P* < 0.05 using non-parametric tests or one-way ANOVA was considered to be statistically significant.

## Electronic supplementary material

Supplementary Information
