# Supplementary material for: Salmonella small RNA fragment Sal-1 facilitates bacterial survival in infected cells via suppressing iNOS induction in a microRNA manner
Source: Sci Rep. 2017 Dec 5;7:16979. doi: 10.1038/s41598-017-17205-4 (PMC5717148; doi:10.1038/s41598-017-17205-4)
Supplement: Supplementary file 1 — Supplementary Information [file 41598_2017_17205_MOESM1_ESM.pdf]

Salmonella small RNA fragment Sal-1 facilitates bacterial survival in infected cells via suppressing iNOS induction in a microRNA manner

Chihao Zhao[1,2*], Zhen Zhou[1,2*], Tianfu Zhang[1,2*], Fenyong Liu[3], Chen-Yu Zhang[1,2†], Ke Zen[1,2†] and Hongwei Gu[1,2†]

[1]State Key Laboratory of Pharmaceutical Biotechnology, Nanjing Advanced Institute for Life Sciences, School of Life Sciences, Nanjing University, Nanjing, Jiangsu, 210046, China. [2]Jiangsu Engineering Research Center for MicroRNA Biology and Biotechnology, Nanjing, Jiangsu 210093, China; [3]School of Public Health, University of California at Berkeley, Berkeley, CA 94720, USA.

Running title: *Salmonella* Sal-1 targets cellular iNOS

Keywords: *Salmonella;* non-coding RNA; iNOS; nitric oxide; infection; miRNA.

*These authors contributed equally to this work

†Correspondences:

Hongwei Gu, PhD

Email: hongweigu@nju.edu.cn

Ke Zen, PhD

Email: kzen@nju.edu.cn

Chen-Yu Zhang, PhD, MD

Email: cyzhang@nju.edu.cn

Supplementary Table S1. Mouse iNOS site mutation.

Name	Sequence (5’→3’)*
Mouse iNOS WT	ATGGCTTGCCCCTGGAAGTTTCTCTTCAAAGTCAAATCCTACCAAAGTGACCTGAAAGAGGAAAAGGA<br>CATTAACAACAACGTGAAGAAAACCCCTTGTGCTGTTCTCAGCCCAACAATACAAGATGACCCTAAGAG<br>TCACCAAAATGGCTCCCCGCAGCTCCTCACTGGGACAGCACAGAATGTTCCAGAATCCCTGGACAAGC<br>TGCATGTGACATCGACCCGTCCACAGTATGTGAGGATCAAAAACTGGGGCAGTGGAGAGATTTTGCATG<br>ACACTCTTCACCACAAGGCCACATCGGATTTCACTTGCAAGTCCAAGTCTTGCTTGGGGTCCATCATGA<br>ACCCCAAGAGTTTGACCAGAGGACCCAGAGACAAGCCTACCCCTCTGGAGGAGCTCCTGCCTCATGCC<br>ATTGAGTTCATCAACCAGTATTATGGCTCCTTTAAAGAGGCAAAAATAGAGGAACATCTGGCCAGGCTG<br>GAAGCTGTAACAAAGGAAATAGAAACAACAGGAACCTACCAGCTCACTCTGGATGAGCTCATCTTTGC<br>CACCAAGATGGCCTGGAGGAATGCCCCTCGCTGCATCGGCAGGATCCAGTGGTCCAACCTGCAGGTCTT<br>TGACGCTCGGAACTGTAGCACAGCACAGGAAATGTTTCAGCACATCTGCAGACACATACTTTATGCCAC<br>CAACAATGGCAACATCAGGTCGGCCATCACTGTGTTCCCCCAGCGGAGTGACGGCAAACATGACTTCA<br>GGCTCTGGAATTCACAGCTCATCCGGTACGCTGGCTACCAGATGCCCGATGGCACCATCAGAGGGGATG<br>CTGCCACCTTGGAGTTCACCCAGTTGTGCATCGACCTAGGCTGGAAGCCCCGCTATGGCCGCTTTGATG<br>TGCTGCCTCTGGTCTTGCAAGCTGATGGTCAAGATCCAGAGGTCTTTGAAATCCCTCCTGATCTTGTGTT<br>GGAGGTGACCATGGAGCATCCCAAGTACGAGTGGTTCCAGGAGCTCGGGTTGAAGTGGTATGCACTGC<br>CTGCCGTGGCCAACATGCTACTGGAGGTGGGTGGCCTCGAATTCCCAGCCTGCCCCTTCAATGGTTGGT<br>ACATGGGCACCGAGATTGGAGTTCGAGACTTCTGTGACACACAGCGCTACAACATCCTGGAGGAAGTG<br>GGCCGAAGGATGGGCCTGGAGACCCACACACTGGCCTCCCTCTGGAAAGACCGGGCTGTCACGGAGAT<br>CAATGTGGCTGTGCTCCATAGTTTCCAGAAGCAGAATGTGACCATCATGGACCACCACACAGCCTCAGA<br>GTCCTTCATGAAGCACATGCAGAATGAGTACCGGGCCCGTGGAGGCTGCCCGGCAGACTGGATTTGGCT<br>GGTCCCTCCAGTGTCTGGGAGCATCACCCCTGTGTTCCACCAGGAGATGTTGAACTATGTCCTATCTCCA<br>TTCTACTACTACCAGATCGAGCCCTGGAAGACCCACATCTGGCAGAATGAGAAGCTGAGGCCCAGGAG<br>GAGAGAGATCCGATTTAGAGTCTTGGTGAAAGTGGTGTTCTTTGCTTCCATGCTAATGCGAAAGGTCAT<br>GGCTTCACGGGTCAGAGCCACAGTCCTCTTTGCTACTGAGACAGGGAAGTCTGAAGCACTAGCCAGGG<br>ACCTGGCCACCTTGTTCAGCTACGCCTTCAACACCAAGGTTGTCTGCATGGACCAGTATAAGGCAAGCA<br>CCTTGGAAGAGGAGCAACTACTGCTGGTGGTGACAAGCACATTTGGGAATGGAGACTGTCCCAGCAAT<br>GGGCAGACTCTGAAGAAATCTCTGTTCATGCTTAGAGAACTCAACCACACCTTCAGGTATGCTGTGTTT<br>GGCCTTGGCTCCAGCATGTACCCTCAGTTCTGCGCCTTTGCTCATGACATCGACCAGAAGCTGTCCCAC<br>CTGGGAGCCTCTCAGCTTGCCCCAACAGGAGAAGGGGACGAACTCAGTGGGCAGGAGGATGCCTTCC<br>GCAGCTGGGCTGTACAAACCTTCCGGGCAGCCTGTGAGACCTTTGATGTACGAAGCAAACATCACATTC<br>AGATCCCGAAACGCTTCACTTCCAATGCAACATGGGAGCCACAGCAATATAGGCTCATCCAGAGCCCGG<br>AGCCTTTAGACCTCAACAGAGCCCTCAGCAGCATCCATGCAAAGAATGTGTTTACCATGAGGCTGAAAT<br>CCCAGCAGAATCTGCAGAGTGAAAAGTCCAGCCGCACCACCCTCCTCGTTCAGCTCACCTTCGAGGGC<br>AGCCGAGGGCCCAGCTACCTGCCTGGGGAACACCTGGGGATCTTCCCAGGCAACCAGACCGCCCTGGT<br>GCAGGGAATCTTGGAGCGAGTTGTGGATTGTCCTACACCACACCAAACTGTGTGCCTGGAGGTTCTGGA<br>TGAGAGCGGCAGCTACTGGGTCAAAGACAAGAGGCTGCCCCCCTGCTCACTCAGCCAAGCCCTCACCT<br>ACTTCCTGGACATTACGACCCCTCCCACCCAGCTGCAGCTCCACAAGCTGGCTCGCTTTGCCACGGACG<br>AGACGGATAGGCAGAGATTGGAGGCCTTGTGTCAGCCCTCAGAGTACAATGACTGGAAGTTCAGCAAC<br>AACCCCACGTTCCTGGAGGTGCTTGAAGAGTTCCCTTCCTTGCATGTGCCCGCTGCCTTCCTGCTGTCG<br>CAGCTCCCTATCTTGAAGCCCCGCTACTACTCCATCAGCTCCTCCCAGGACCACACCCCCTCGGAGGTTC<br>ACCTCACTGTGGCCGTGGTCACCTACCGCACCCGAGATGGTCAGGGTCCCCTGCACCATGGAGTCTGCA<br>GCACTTGGATCAGGAACCTGAAGCCCCAGGACCCAGTGCCCTGCTTTGTGCGAAGTGTCAGTGGCTTC<br>CAGCTCCCTGAGGACCCCTCCCAGCCTTGCATCCTCATTGGGCCTGGTACGGGCATTGCTCCCTTCCGA<br>AGTTTCTGGCAGCAGCGGCTCCATGACTCCCAGCACAAAGGGCTCAAAGGAGGCCGCATGAGCTTGGT<br>GTTTGGGTGCCGGCACCCGGAGGAGGACCACCTCTATCAGGAAGAAATGCAGGAGATGGTCCGCAAGA<br>GAGTGCTGTTCCAGGTGCACACAGGCTACTCCCGGCTGCCCGGCAAACCCAAGGTCTACGTTCAGGAC<br>ATCCTGCAAAAGCAGCTGGCCAATGAGGTACTCAGCGTGCTCCACGGGGAGCAGGGCCACCTCTACAT<br>TTGCGGAGATGTGCGCATGGCTCGGGATGTGGCTACCACGTTGAAGAAGCTGGTGGCCACCAAGCTGA<br>ACTTGAGCGAGGAGCAGGTGGAAGACTATTTCTTCCAGCTCAAGAGCCAGAAACGTTATCATGAAGATA<br>TCTTCGGTGCAGTCTTTTCCTATGGGGCAAAAAAGGGCAGCGCCTTGGAGGAGCCAAAAGCCACGAGG<br>CTCTGA
Mouse iNOS MUT	atggcttgccccctggaagtttctcttcaaagtcaaatcctaccaaagtgacctgaaagaggaaaaggacattaacaacaacgtg<br>aagaaaaccccttgtgctgttctcagcccaacaatacaagatgaccctaagagtcaccaaaatggctccccgcagctcctcact<br>gggacagcacagaatgttccagaatccctggacaagctgcatgtgacatcgacccgtccacagtatgtgaggatcaaaaactg<br>gggcagtggagagattttgcatgacactcttcaccacaaggccacatcggatttcacttgcaaAtcGaagtcAtgcCtTggg<br>AGTatcatgaaccccaagagtttgaccagaggacccagagacaagcctacccctctggaggagctcctgcctcatgccatt<br>gagttcatcaaccagtattatggctcctttaaagaggcaaaaatagaggaacatctggccaggctggaagctgtaacaaaggaa<br>atagaaacaacaggaacctaccagctcactctggatgagctcatctttgccaccaagatggcctggaggaatgcccctcgctg<br>catcggcaggatccagtggtccaacctgcaggtctttgacgctcggaactgtagcacagcacaggaaatgtttcagcacatctg<br>cagacacatactttatgccaccaacaatggcaacatcaggtcggccatcactgtgttcccccagcggagtgacggcaaacatg<br>acttcaggctctggaattcacagctcatccggtacgctggctaccagatgcccgatggcaccatcagaggggatgctgccacc<br>ttggagttcacccagttgtgcatcgacctaggctggaagccccgctatggccgctttgatgtgctgcctctggtcttgcaagctga<br>tggtcaagatccagaggtctttgaaatccctcctgatcttgtgttggaggtgaccatggagcatcccaagtacgagtggttccag<br>gagctcgggttgaagtggtatgcactgcctgccgtggccaacatgctactggaggtgggtggcctcgaattcccagcctgccc<br>cttcaatggttggtacatgggcaccgagattggagttcgagacttctgtgacacacagcgctacaacatcctggaggaagtggg<br>ccgaaggatgggcctggagacccacacactggcctccctctggaaagaccgggctgtcacggagatcaatgtggctgtgctc<br>catagtttccagaagcagaatgtgaccatcatggaccaccacacagcctcagagtccttcatgaagcacatgcagaatgagtac<br>cgggcccgtggaggctgcccggcagactggatttggctggtccctccagtgtctgggagcatcacccctgtgttccaccagga<br>gatgttgaactatgtcctatctccattctactactaccagatcgagccctggaagacccacatctggcagaatgagaagctgagg<br>cccaggaggagagagatccgatttagagtcttggtgaaagtggtgttctttgcttccatgctaatgcgaaaggtcatggcttcacg<br>ggtcagagccacagtcctctttgctactgagacagggaagtctgaagcactagccagggacctggccaccttgttcagctacg<br>ccttcaacaccaaggttgtctgcatggaccagtataaggcaagcaccttggaagaggagcaactactgctggtggtgacaagc<br>acatttgggaatggagactgtcccagcaatgggcagactctgaagaaatctctgttcatgcttagagaactcaaccacaccttca<br>ggtatgctgtgtttggccttggctccagcatgtaccctcagttctgcgcctttgctcatgacatcgaccagaagctgtcccacctg<br>ggagcctctcagcttgccccaacaggagaaggggacgaactcagtgggcaggaggatgccttccgcagctgggctgtacaa<br>accttccgggcagcctgtgagacctttgatgtacgaagcaaacatcacattcagatcccgaaacgcttcacttccaatgcaacat

gggagccacagcaatataggctcatccagagcccggagcctttagacctcaacagagccctcagcagcatccatgcaaagaa
tgtgtttaccatgaggctgaaatcccagcagaatctgcagagtgaaaagtccagccgcaccaccctcctcgttcagctcaccttc
gagggcagccgagggcccagctacctgcctggggaacacctggggatcttcccaggcaaccagaccgccctggtgcaggg
aatcttggagcgagttgtggattgtcctacaccacaccaaactgtgtgcctggaggttctggatgagagcggcagctactgggt
caaagacaagaggctgccccccctgctcactcagccaagccctcacctacttcctggacattacgacccctcccacccagctgc
agctccacaagctggctcgctttgccacggacgagacggataggcagagattggaggccttgtgtcagccctcagagtacaat
gactggaagttcagcaacaaccccacgttcctggaggtgcttgaagagttcccttccttgcatgtCccGgctgccttcctgctgt
cgcagctccctatcttgaagccccgctactactccatcagctcctcccaggaccacacccccctcggaggttcacctcactgtgg
ccgtggtcacctaccgcacccgagatggtcagggtcccctgcaccatggagtctgcagcacttggatcaggaacctgaagcc
ccaggacccagtgccctgctttgtgcgaagtgtcagtggcttccagctccctgaggacccctcccagccttgcatcctcattggg
cctggtacgggcattgctcccttccgaagtttctggcagcagcggctccatgactcccagcacaaagggctcaaaggaggcc
gcatgagcttggtgtttgggtgccggcacccggaggaggaccacctctatcaggaagaaatgcaggagatggtccgcaaga
gagtgctgttccaggtgcacacaggctactcccggctgcccggcaaacccaaggtctacgttcaggacatcctgcaaaagca
gctggccaatgaggtactcagcgtgctccacggggagcagggccacctctacatttgcggagatgtgcgcatggctcgggat
gtggctaccacgttgaagaagctggtggccaccaagctgaacttgagcgaggagcaggtggaagactatttcttccagctcaa
gagccagaaacgttatcatgaagatatcttcggtgcagtcttttcctatggggcaaaaaagggcagcgccttggaggagccaaa
agccacgaggctctga

*All site mutations are shown in capital and marked with green in mouse iNOS mut .

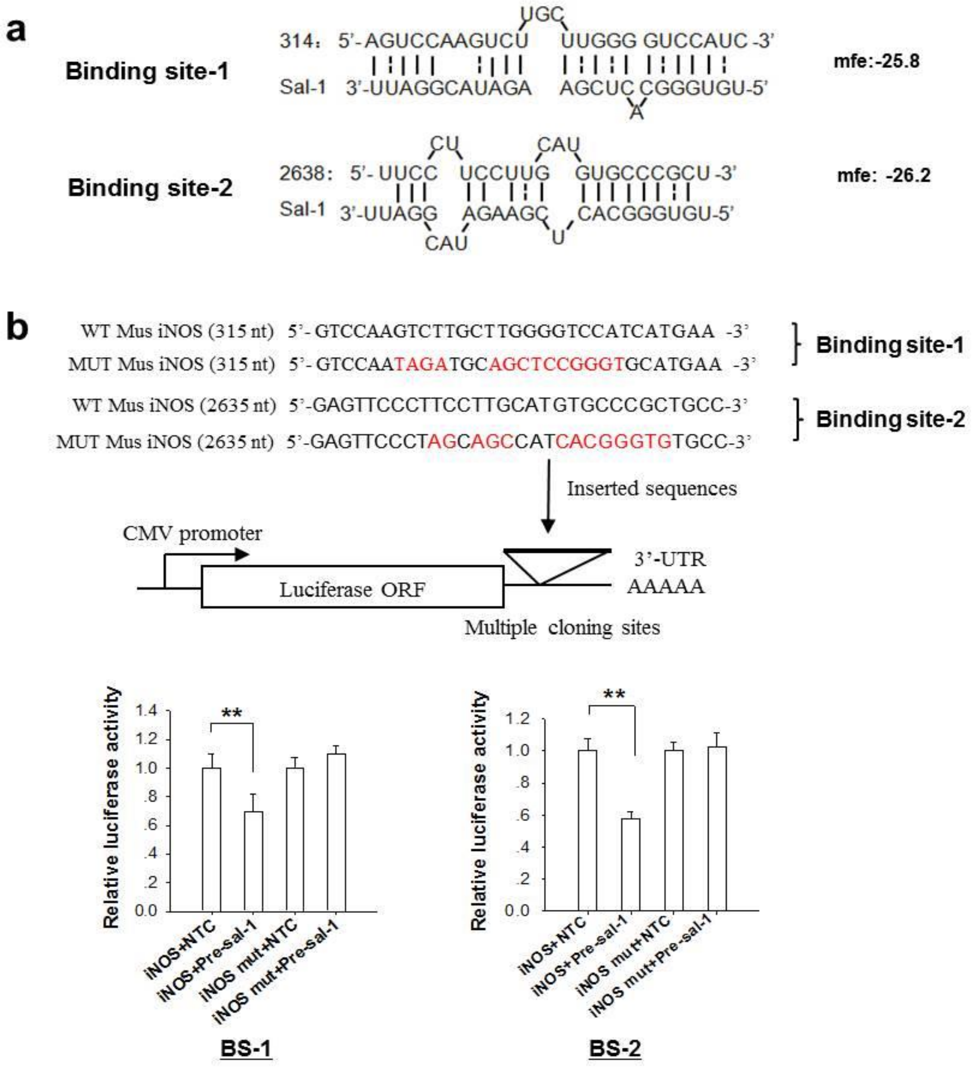

Figure S1. Sal-1 targets mouse iNOS mRNA. (a) Sal-1 binding sites on mouse iNOS mRNA analysed by the RNAhybrid software. Two binding sites were also mutated, respectively. (b) Luciferase reporter assay confirmed that Sal-1 specifically bound to the binding site 1 and 2. The data are presented as the mean ± SEM (n=3). **, $P<0.01$.

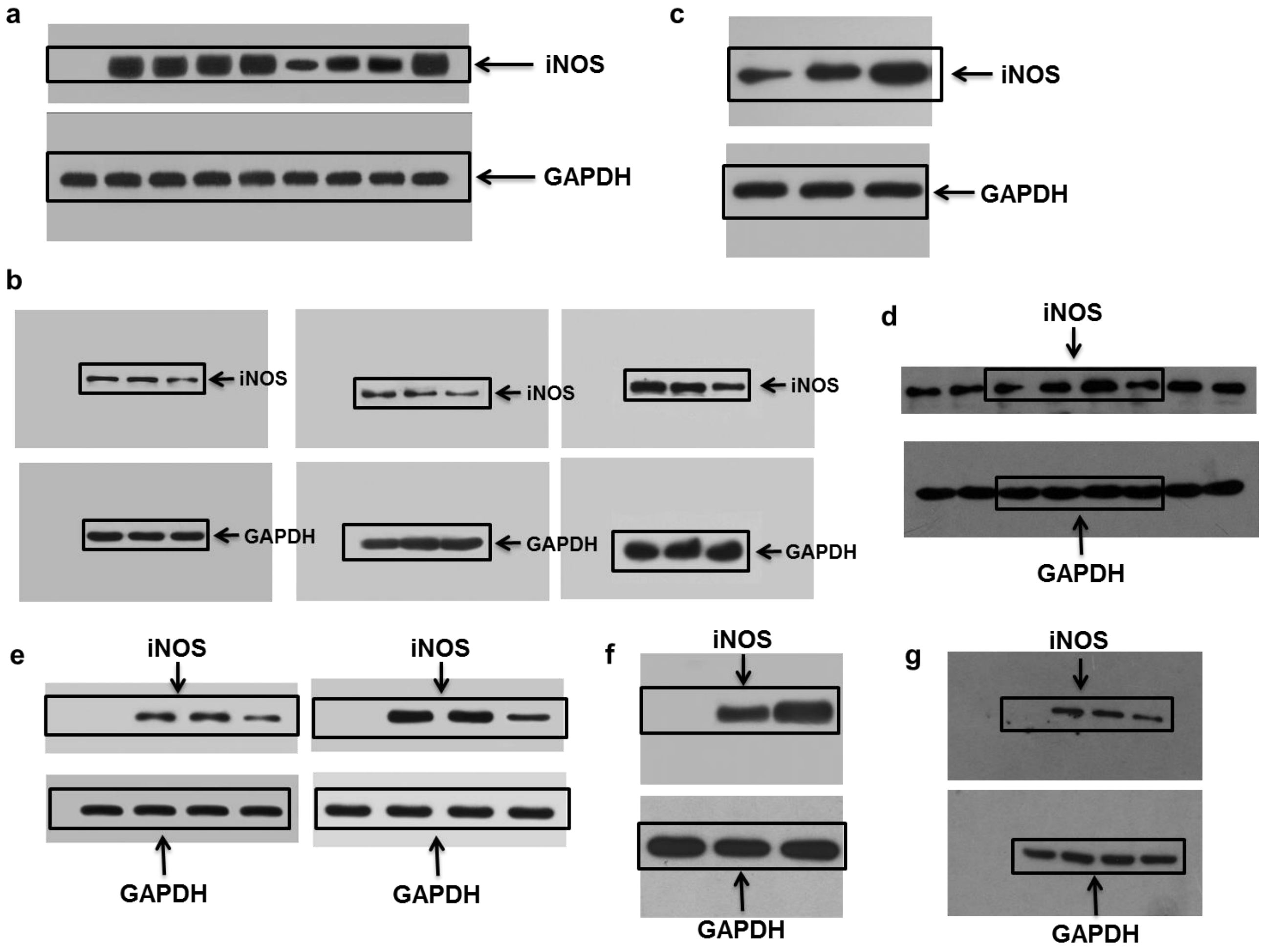

Figure S2. Full gel images of western bolts. (a) The full-size images corresponding to those shown in Figure 2d, (b) Figure 3a, (c) Figure 3c, (d) Figure 4a, (e) Figure 5a, (f) Figure 5d and (g) Figure 5g.
